# Efficacy of Systemic Treatments of Nail Psoriasis: A Systemic Literature Review and Meta-Analysis

**DOI:** 10.3389/fmed.2021.620562

**Published:** 2021-02-10

**Authors:** Xuan Zhang, Bingbing Xie, Yanling He

**Affiliations:** ^1^Department of Dermatology, Beijing Chao-Yang Hospital, Capital Medical University, Beijing, China; ^2^Department of Pulmonary and Critical Care Medicine, China-Japan Friendship Hospital, Beijing, China

**Keywords:** nail, psoriasis, systemic treatments, systemic review, meta-analysis

## Abstract

**Importance:** Nail involvement is a common condition in patients with psoriasis. The treatment of nail psoriasis is considered challenging and is often left untreated by physicians.

**Objective:** To assess the efficacy of current systemic treatments on nail psoriasis.

**Data Sources:** PubMed, EMBASE, and Cochrane Central Register of Controlled Trials (CENTRAL) were searched for relevant articles from inception to September 1, 2020. Included articles were restricted to English language and human studies.

**Study Selection:** This was a systematic literature review with meta-analysis. Thirty-five random control trials that evaluated systemic therapies for nail psoriasis were selected in the systemic review. Among them, we retained 14 trials for meta-analysis.

**Data Extraction and Synthesis:** This study was conducted in accordance with the preferred reporting items for systematic review and meta-analysis protocols (PRISMA-P) 2015 statement. All steps were performed by two independent investigators, and any disagreements were resolved by a third investigator. Meta-analysis of aggregated study data was conducted to assess therapeutic efficacy. The use of random-effects model was based on high heterogeneity as a variable endpoint in different studies.

**Main Outcomes and Measures:** Therapeutic effects on nail psoriasis were expressed in terms of effect sizes with 95% CIs.

**Results:** We included 35 random control trials (RCTs) in this systemic review. At baseline, a high prevalence (62.1%) of nail psoriasis was confirmed. The meta-analysis included 14 trials highlighting that biologic and small-molecule therapies were effective in treating nail psoriasis with variable effect size magnitudes [−0.89 (−1.10, −0.68), *I*^2^ = 84%]. In particular, tofacitinib and ixekizumab showed the most significant scale of effect size magnitudes in treating nail psoriasis (−1.08 points and −0.93 points, respectively). We also found that a higher dose of tofacitinib and ixekizumab had similar effectiveness, and anti-IL-17 agents seem to be superior in effectiveness compared to anti-TNF-α therapies in the treatment of nail psoriasis. However, these results must be displayed carefully as variable endpoints in different studies.

**Conclusions and Relevance:** This study provides a comprehensive overview of systemic treatments for nail psoriasis. For patients with psoriatic nail damage who are candidates of systemic therapies, the priority should be given to administering biologic and small-molecule therapies, especially anti-IL-17 drugs.

## Introduction

Psoriasis is a chronic systemic inflammatory disease that frequently affects the nails. Approximately 40–50% of patients with psoriasis have concurrent nail involvement, with a lifetime incidence of 80–90% (1, 2). Nail psoriasis is associated with pain, cosmetic problems, and impaired finger function, with remarkably negative effects on the patient's quality of life (3, 4). Nail involvement in patients with psoriasis is considered a predictor for the development of psoriatic arthritis (5). High-resolution magnetic resonance imaging (MRI) showed that the integral supporting structure of the nail is formed by extensor tendon enthesis (6). Through this anatomical link between the nail and the joint, inflammatory responses at the affected joint in patients with psoriatic arthritis (PsA) often extend to the nail bed, suggesting that psoriatic nails can be considered as the tip of the iceberg of systemic inflammation (7). Based on this, nail psoriasis is often resistant to conventional treatments, such as topical and intralesional therapies, which are targeting at local inflammation response. Moreover, the structure of the nail presents therapeutic challenges, such as poor penetration of topical therapy across the nail plate and pain associated with intralesional therapies (8, 9). Furthermore, it has been reported that nail psoriasis promptly recurs once patients halt local therapies (10–12).

Nail psoriasis has a wide spectrum of clinical manifestations, depending on the part of the affected structure, which can be divided into the nail matrix (pitting, leukonychia, red spots in the lunula, and nail plate crumbling) or nail bed (oil drop discoloration, onycholysis, nail bed hyperkeratosis, and splinter hemorrhage) (8). In addition to a clinical description of improvement or exacerbation of nail psoriasis features, there are severity scoring systems, including the Nail Psoriasis Severity Index (NAPSI), Nail Area Severity (NAS), and Psoriasis Nail Severity Score (PNSS).

In recent years, a significant alleviation of psoriatic nails has been reported with the widespread use of small-molecule therapies and biologic agents for cutaneous psoriasis (13). Therefore, this study aimed at providing a systematic review and meta-analysis on the effectiveness of systemic therapies that are currently available for patients with psoriatic nails.

## Materials and Methods

We conducted a systematic review and meta-analysis of random control trials for the evaluation of treatments for nail psoriasis. This study was conducted in accordance with the preferred reporting items for systematic review and meta-analysis protocols (PRISMA-P) 2015 statement (14). It is also registered in PROSPERO (https://www.crd.york.ac.uk/prospero/; registration number CRD42020204238).

### Literature Search

A computer-based literature search was performed to identify relevant articles published from inception to September 1, 2020, in PubMed, EMBASE, and the Cochrane Central Register of Controlled Trials (CENTRAL). The main search terms were “*psoriasis*” and “*nail*.” Vocabulary and syntax were adapted for each database. The literature search was restricted to English language and human studies. In addition, the references of these articles were also screened for relevant articles, and clinical trials registered at ClinicalTrials.gov were searched for details of relevant trials.

### Study Selection

The inclusion and exclusion criteria were determined before the search. The included studies fulfilled the following inclusion criteria: (1) study design was limited to RCT; (2) the study participants should be adults (age > 18 years) with a diagnosis of any type of psoriasis without any other nail disorder; (3) the evaluated interventions were restricted to traditional systemic immunomodulating agents, small-molecule therapies, and biologic agents; (4) severity scoring systems should be used to evaluate the involvement of nail psoriasis at baseline and at the end of study or the improvement of psoriatic nail during the treatment phase.

### Data Abstraction and Quality Assessment

Two independent reviewers abstracted data using a predefined data extraction form. The following information was extracted from each study: author, year of publication, design of study, blind time period, patient type, details of the interventions, sample size, baseline nail psoriasis involvement, and the improvement at each visit till the end of study. We independently assessed the quality of each included study in accordance with the Cochrane handbook of systematic reviews of interventions 5.2, which covers the following: (1) random sequence generation (selection bias); (2) allocation concealment (selection bias); (3) blinding of participants and treatment providers (performance bias); (4) blinding of outcome assessors (detection bias), (5) incomplete outcome data (attrition bias), (6) selective reporting (reporting bias), and (7) other biases. Disagreements over any risk of bias in particular studies were resolved by a third reviewer.

### Statistical Analysis

We performed statistical analyses using the Review Manager V5.3 (The Nordic Cochrane Center, The Cochrane Collaboration) and STATA V15.0 (StataCorp). The identified studies used severity scoring systems in the range 0–8 to 0–160; thus, scores will be scaled down to range 0–8 for meta-analysis for aggregation across the trials. We applied the mean difference (MD) with 95% CIs as the change in psoriatic nail involvement. The reduction in the scores over the observation period indicated an improvement in nail psoriasis. We used the random effect model to pool data to evaluate the overall effect. Heterogeneity was assessed using the *I*^2^ statistic. The possibility of publication bias was assessed using a funnel plot and Egger test. Some trials included more than one intervention group, for which the control groups were equalized among the intervention groups.

## Results

### Systematic Review

**Figure 1:**
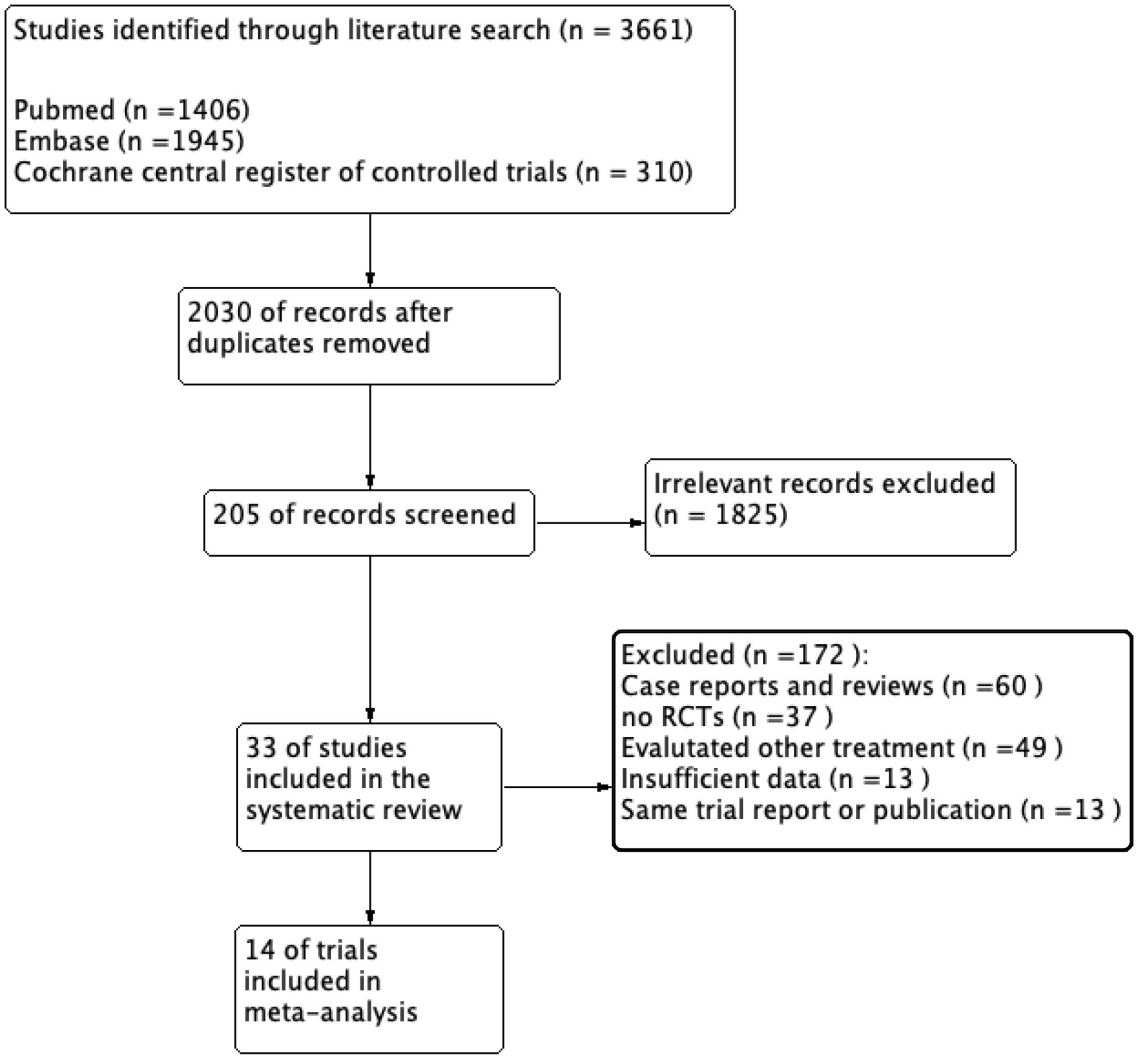
Flow chart of Search Strategy and Study Selection.

We identified 2,030 articles matching the search criteria after removing duplicate publications. We extracted 1,825 articles after reading the title or abstract. Furthermore, we retained 33 articles after a full-text review. The results of two different trials were presented in two articles (15, 16). Thus, we included 35 trials in the systematic review. In addition, four trials (17–20) did not mention the portion of nail involvement or enrolled patients with nail psoriasis, the remaining 31 trials included 17,254 patients with psoriasis, and 10,720 (62.1%) had nail involvement. The flow diagram is shown in Figure 1, and Supplementary Figure 1 provides the quality assessment for the included trials.

#### PDE4 Inhibitor: Apremilast (3 Trials)

In a placebo-controlled study on 266 patients, Paul et al. (21) reported that apremilast resulted in a trend of greater percentage reduction in NAPSI score vs. placebo (29.0 vs. 7.1%, *P* = 0.052) at week 16. Papp et al. (22) compared apremilast with placebo in 558 patients with nail psoriasis. They demonstrated that apremilast significantly reduced the activity of nail psoriasis after a treatment period of 16 weeks, whereas placebo had no effect (*P* < 0.0001). However, Reich et al. (23) studied 142 patients to assess the efficacy of apremilast and etanercept. Compared with the placebo group (−10.1%), the etanercept group (−37.3%, *P* = 0.002) experienced a significant improvement in NAPSI score, whereas apremilast (−18.7%, *P* = 0.495) had no effect at week 16.

#### JAK Inhibitor: Tofacitinib (3 Trials)

Merola et al. (16) pooled data from 2 placebo-controlled studies (1,018 patients) showing a mean improvement of the NAPSI score (0–80) by 7.9 points in the tofacitinib 5 mg BID group and 10.5 points in the tofacitinib 10 mg BID group compared with the 0.4 points in the placebo group (*p* < 0.001) at week 16. In another (24) study with 266 patients (24), 116 had nail psoriasis. At week 16, the tofacitinib 10 mg BID group produced significantly greater changes in the NAPSI score vs. the placebo group (−33.32 vs. 7.91%, *P* = 0.01). Asahina et al. (25) evaluated the efficacy of different doses of tofacitinib in 66 patients. After 16 weeks of treatment, there were no significant differences in the reduction of NAPSI score between the tofacitinib 5 mg/BID and 10 mg/BID groups (−11.3 vs. −10.2%).

#### Anti-GM-CSF Agent: Namilumab (1 Trial)

Papp et al. (26) compared namilumab to placebo in 122 patients. At the end of 12 weeks of treatment, the alleviation of nail psoriasis evaluated by NAPSI score was −2.5 points and −1.0 points in the namilumab 80 and 150 mg group, respectively, compared with 1.5 points in the placebo group (*P* = 0.05 and 0.121, respectively).

#### Anti-TNF-α Agent

##### Etanercept (1 Trial)

Mease et al. (27) examined the efficacy of methotrexate monotherapy relative to that of etanercept monotherapy and their combination in 588 patients. There was no significant difference in mNAPSI changes between the two monotherapies at week 24, while combining therapy showed a greater decrease in mNAPSI compared with methotrexate monotherapy (−1.7 vs. −1.1, *P* = 0.02).

##### Adalimumab (2 Trials)

Elewski et al. (18) compared adalimumab with placebo in 217 patients, demonstrating that adalimumab induced greater improvement in the quality of life of patents with nail psoriasis. Significant improvement in the NAPSI score was as early as week 8 in 18.8% for the adalimumab group and 3.5% for the placebo group (*P* < 0.01). Leonardi et al. (28) compared adalimumab vs. placebo in 72 patients. The mean percentage improvement in NAPSI score was significantly greater for adalimumab than for placebo (50 vs. 8%, *P* = 0.02) at week 16.

##### Infliximab (2 Trials)

In a study by Reich et al. (29) with 378 patients, 80.7% of patients had a psoriatic nail with a mean NAPSI score of 4.53 at baseline. The mean change in the NAPSI score was 26.0% at week 10 and 56.3% at week 24 in the infliximab group compared with −5.6 and −3.2% in the placebo group (*p* < 0.0001), respectively. In another study (30) of 43 patients, infliximab-treated patients achieved a higher reduction in NAPSI score (0–8) compared with placebo-treated patients (1.4 vs. −0.3), as early as week 10.

##### Certolizumab Pegol (1 Trial)

Mease et al. (31) included 409 patients with PsA treated with certolizumab pegol vs. placebo. We recorded 73.3% of patients with baseline nail disease, and after a treatment period of 24 weeks, mNAPSI (0–8) changed from baseline was −1.6 for the certolizumab pegol 200 mg Q2W group and −2.0 for the certolizumab pegol 400 mg Q4W group compared with −1.1 for the placebo group (*p* = 0.003 and *p* < 0.001, respectively).

##### Golimumab (3 Trials)

Kavanaugh et al. (32) used golimumab vs. placebo on 405 patients with PsA. The median improvement in NAPSI score from baseline to weeks 14 and 24 was significantly greater (*P* < 0.001) in the golimumab 50 mg group (25, 43%) and the golimumab 50 mg group (33, 54%) compared to that in the placebo group (0, 0%, respectively). Vieira-Sousa et al. (20) evaluated methotrexate monotherapy or combination therapy with golimumab in 44 patients. After 12 weeks of treatment, the medium percentage of reduction in target fingernail NAPSI score (0–8) from baseline for combination therapy was greater than that of methotrexate monotherapy (−2 vs. 0, *P* = 0.044). Mease et al. (33) compared golimumab vs. placebo in 367 patients. In this study, they observed a discernible clinical benefit in alleviating nail psoriasis for golimumab through 14 weeks of treatment (−9.6 vs. 1.9, *P* < 0.001).

##### Brodalumab (1 Trial)

Elewski et al. (34) pooled two trials to evaluate the efficacy of brodalumab compared with that of ustekinumab in 593 patients with nail psoriasis. Among these, 283 had nail involvement. At week 52, 63.8% of patients achieved NAPSI = 0 for the brodalumab group vs. 39.1% for the ustekinumab group (*P* < 0.05).

#### Anti-IL-23 Agent

##### Ustekinumab (2 Trials)

Rich et al. (35) compared ustekinumab vs. placebo during 12 weeks of treatment in 766 patients. Treatment with ustekinumab 45 or 90 mg resulted in significantly better percentage improvement in NAPSI score than the placebo group (*P* < 0.001 and *P* = 0.001, respectively). However, Igarashi et al. (36) reported that there was no significant NAPSI improvement in ustekinumab 45 and 90 mg groups vs. placebo at week 12.

##### Guselkumab (2 Trials)

Ohtsuki et al. (37) compared guselkumab with placebo in 192 patients. Among patients with nail psoriasis (*n* = 126), a significant decrease in mNAPSI score (0–8) of −1.2 and −1.5 was observed for the guselkumab 50 and 100 mg groups, compared with −0.2 for the placebo group, at week 16. Foley et al. (38) pooled two studies comparing guselkumab and adalimumab to placebo in 928 patients with fingernail psoriasis. The mean improvements in target NAPSI score were significantly greater for the treatment group (37.5 and 41.70%, respectively) than for the placebo group (0.7%; *P* < 0.001) at week 16.

#### Anti-IL-17 Agent

##### Secukinumab (3 Trials)

Reich et al. (17) compared secukinumab vs. placebo in 198 patients during week 16. Treatment with secukinumab resulted in significant improvements in nail psoriasis compared with placebo (*P* < 0.001); NAPSI improvements were −45.3, −37.9, and −10.8% for secukinumab 300 and 150 mg and placebo, respectively. Further alleviation of psoriatic nails was shown by week 32: NAPSI change from baseline was −63.2% for secukinumab 300 mg and −52.6% for secukinumab 150 mg. Two placebo-controlled studies (39, 40) evaluated the effectiveness of secukinumab in nail psoriasis. The mean changes in NAPSI were significantly greater for secukinumab than for the placebo group (*P* < 0.0001).

##### Ixekizumab (7 Trials)

In a placebo-controlled study with 58 patients, Leonardi et al. (41) highlighted that 75 mg/150 mg q4w ixekizumab markedly alleviated the clinical symptoms of nail psoriasis compared with the placebo group as early as week 2. The SPIRIT-P1 study (42) compared ixekizumab with adalimumab and placebo in 417 patients. Among them, 289 had nail psoriasis. At week 24, the mean changes from baseline in the NAPSI score were significantly greater for the ixekizumab q4w (−14.0), ixekizumab q2w (−15.5), and adalimumab (−10.7) groups than for the placebo group (−2.4) (*p* < 0.001). A head-to-head trial (43) of 189 patients with nail psoriasis revealed a significantly greater number of patients achieved NAPSI = 0 with ixekizumab vs. ustekinumab as early as week 16. The UNCOVER-1 study (15) compared ixekizumab (80 mg q2w, 80 mg q4w) to placebo in 847 patients. The mean improvements in the NAPSI (0–80) were 7.24, 7.19, and −2.17 points, respectively (*p* < 0.001) at week 12. The UNCOVER-2 study (15) compared the same two doses of ixekizumab with etanercept (50 mg twice a week) and placebo in 751 patients. Treatment with ixekizumab 80 mg q2w or q4w resulted in an equivalent reduction in the NAPSI score (8.6 and 7.39, respectively), which was significantly better than that of patients treated with etanercept (5.34 points) and placebo (0.82 points, *P* < 0.001). Kerkhof et al. (44) performed a *post-hoc* analysis of the UNCOVER-3 study on 809 patients with baseline fingernail psoriasis comparing the efficacy of ixekizumab with etanercept and placebo. Ixekizumab provided significant improvement in fingernail NAPSI score as early as week 2 vs. etanercept (5.1 vs −7.9%, *P* = 0.024). At week 12, greater mean NAPSI improvements were achieved in the ixekizumab q4w group (36.7%) than in the placebo group (−34.3%, *P* < 0.001) and the etanercept group (20.0%, *P* = 0.048). In a head-to-head trial with 368 nail psoriasis patients, Mease et al. (45) compared ixekizumab with adalimumab. After 24 weeks of treatment, the mean change from baseline NAPSI was −15.89 for the ixekizumab group vs. −12.53 for the adalimumab group (*P* = 0.001).

#### Traditional Systemic Immunomodulating Treatments (3 Trials)

Reich et al. (46) compared alitretinoin to placebo in 31 patients with palmoplantar pustulosis. The changes from baseline in the NAPSI score were similar for the alitretinoin and the placebo groups at weeks 12 and 24. Warren et al. (47) enrolled 120 patients to evaluate the efficacy of subcutaneous methotrexate in treating nail psoriasis. At week 16, there were no significant (*P* = 0.40) changes in NAPSI scores between the methotrexate group and the placebo group. Gümüşel et al. (19) enrolled 17 patients with nail psoriasis to compare the effectiveness of methotrexate and cyclosporine. After 24 weeks of treatment, the reduction of the NAPSI score from baseline was 43.3 and 37.2% for the methotrexate and cyclosporine groups, respectively.

The summary of systemic treatments for nail psoriasis are provided in Supplementary Table 1.

### Meta-Analysis

Among the trials selected for the systematic review, we included 14 trials that provided the outcome measurement of the alleviation of nail psoriasis between baseline and the end of the study. The characteristics of the selected trials are summarized in Table 1.

**Table 1:** Characteristics of the 14 Included Studies for meta-analysis.

**Reference**	**NCT**	**Treatment**	**Design**	**Patients**	**Outcome measure of nail psoriasis**
**Placebo control trials**					
(41)	NCT01107457	150 mg of ixekizumab at 0, 2, 4, 8, 12, and 16 weeks	Parallel groups 12 w	Ixekizumab 10 Placebo 15	Total nail NAPSI (0–160)
(15)	NCT01474512	160 mg ixekizumab at baseline followed by 80 mg Q4W or Q2W	Parallel groups 12 w	Ixekizumab Q2W 283 Ixekizumab Q4W 281 Placebo 283	Total fingernail NAPSI (0–80)
(44)	NCT01646177	160 mg ixekizumab at baseline followed by 80 mg Q4W or Q2W etanercept 50 mg twice weekly	Parallel groups 12 w	Ixekizumab Q2W 229 Ixekizumab Q4W 228 Etanercept 236 Placebo 116	Total NAPSI fingernail (0–80)
(15)	NCT01597245	160 mg ixekizumab at baseline followed by 80 mg Q4W or Q2W etanercept 50 mg twice weekly	Parallel groups 12 w	Ixekizumab Q2W 206 Ixekizumab Q4W 215 Etanercept 219 Placebo 111	Total fingernail NAPSI (0–80)
(42)	NCT01695239	Ixekizumab 160 mg at baseline followed by 80 mg Q4W or Q2W INF 40 mg/Q2W	Parallel groups 12 w	Adalimumab Q2W 71 Ixekizumab Q4W 70 Ixekizumab Q2W 74 Placebo 74	Total fingernail mNAPSI (0–80)
(35)	NCT00267969	Ustekinumab 90 mg at weeks 0, 4, 16, and 28	Parallel groups 12 w	Ustekinumab 187 Placebo 176	Target fingernail NAPSI (0–8)
(37)	NCT02325219	Guselkumab 100 mg at weeks 0, 4, and every 8 weeks	Parallel groups 16 w	Guselkumab 40 Placebo 42	Target fingernail NAPSI (0–8)
(16)	NCT01276639	Tofacitinib 5 mg/BID or 10 mg/BID	Parallel groups 16 w	Tofacitinib 5 mg 224 Tofacitinib 10 mg 229 Placebo 102	Total fingernail NAPSI (0–80)
(16)	NCT01309737	Tofacitinib 5 mg/BID or 10 mg/BID	Parallel groups 16 w	Tofacitinib 5 mg 184 Tofacitinib 10 mg 175 Placebo 104	Total fingernail NAPSI (0–80)
(24)	NCT01815424	Tofacitinib 5 mg/BID or 10 mg/BID	Parallel groups 16 w	Tofacitinib 5 mg 38 Tofacitinib 10 mg 40 Placebo 38	Total fingernail NAPSI (0–80)
(30)	-	Infliximab 5 mg/kg at weeks 0, 2, and 6 and every 8 weeks	Parallel groups 14 w	Infliximab 29 Placebo 14	Target fingernail NAPSI (0–8)
(33)	NCT02181673	Golimumab 2 mg/kg at weeks 0 and 4 and every 8 weeks	Parallel groups 14 w	Golimumab 197 Placebo 170	Total fingernail mNAPSI (0–130)
(26)	NCT02129777	Namilumab 80 mg at week 2, 6, and 10 with a loading (double) dose at week 0	Parallel groups 12 w	Namilumab 25 Placebo 24	Total fingernail NAPSI (0–80)
**Head-to-head trial**					
(25)	NCT01519089	Tofacitinib 5 or 10 mg/BID	Parallel groups 16 w	Tofacitinib 5 mg 32 Tofacitinib 10 mg 34	Total fingernail NAPSI (0–80)

*BID, twice a day; Q2W, every 2 weeks; Q4W, every 4 weeks; NAPSI, Nail Psoriasis Severity Index; mNAPSI, modified Nail Psoriasis Severity Index; NCT, ClinicalTrials.gov Identifier*.

#### Efficacy of Treatments

**Figure 2:**
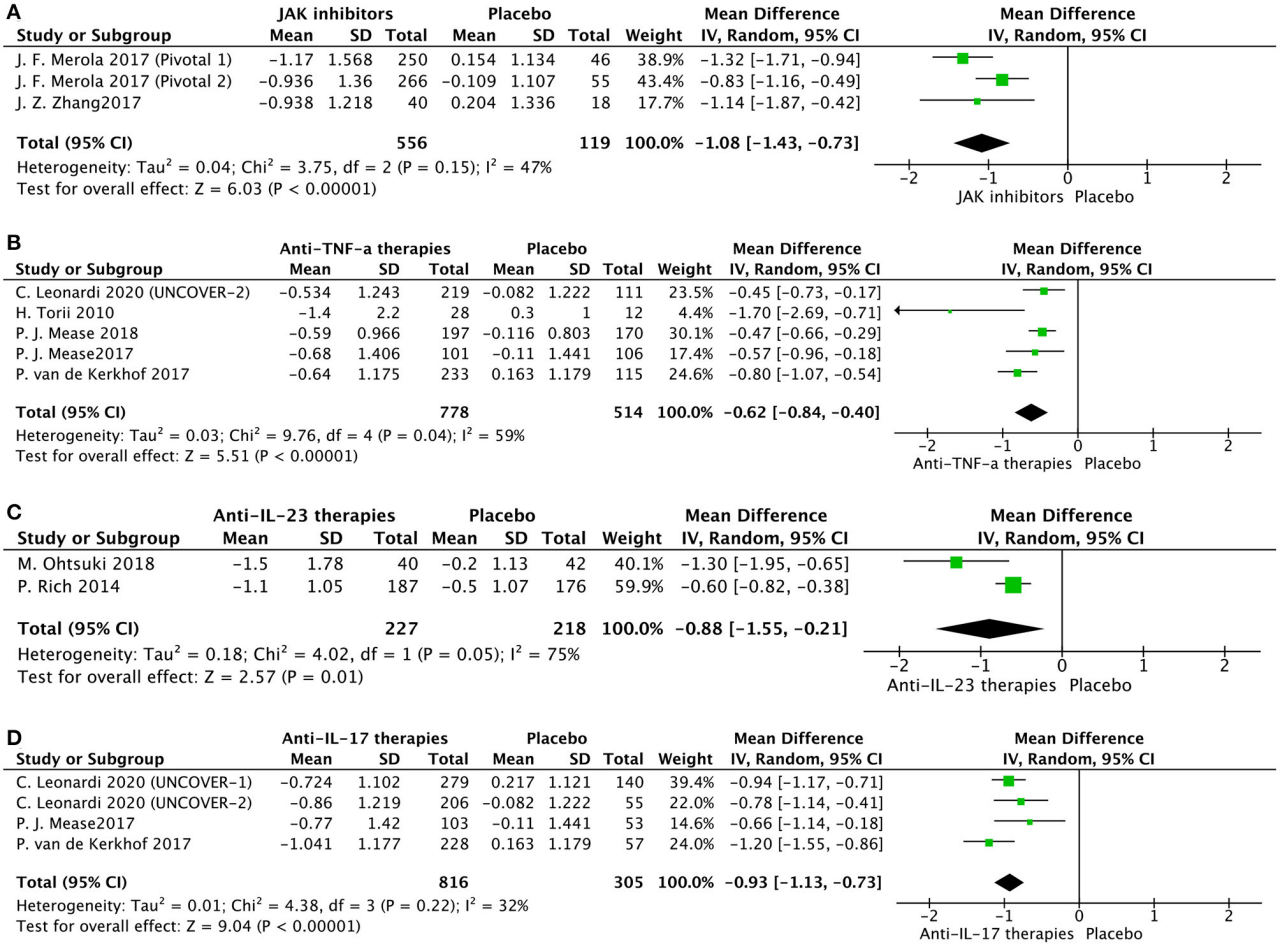
Subgroup meta-analysis comparing the effect of interventions vs. placebo for the treatment of nail psoriasis. **(A)** JAK inhibitors vs. placebo; **(B)** anti-TNF-a agents vs. placebo; **(C)** anti-IL-23 agents vs. placebo; **(D)** anti-IL-17 agents vs. placebo.

We evaluated 13 trials comparing the effectiveness of the interventions with placebo at variable endpoints at week 12 in seven trials (15, 26, 35, 41, 42, and 44), at week 14 in two trials (30, 33), and at week 16 in four trials (16, 24, 37). For some trials comparing different doses of interventions with placebo, the highest dose group was included in the global analysis. Positive comparisons contained in three trials were also included in this meta-analysis. Combined results from included trials were included in this global analysis (Supplementary Figure 2) and comparing interventions with placebo led to a significant decline in mean NAPSI score −0.89 points (95% CI [−1.10, −0.68]; *P* < 0.00001) and highlighted an immense level of heterogeneity (*I*^2^ = 84%). Accordingly, the subgroup analysis of treatment was employed to handle this bias: Figure 2A for JAK inhibitors [tofacitinib (16, 24)], Figure 2B for anti-TNF [etanercept (15, 44), adalimumab (42), infliximab (30) and golimumab (33)], Figure 2C for Anti-IL-23 [ustekinumab (35) and guselkumab (37)], and Figure 2D for Anti-IL-17 [ixekizumab (15, 42, 44)].

**Figure 3:**
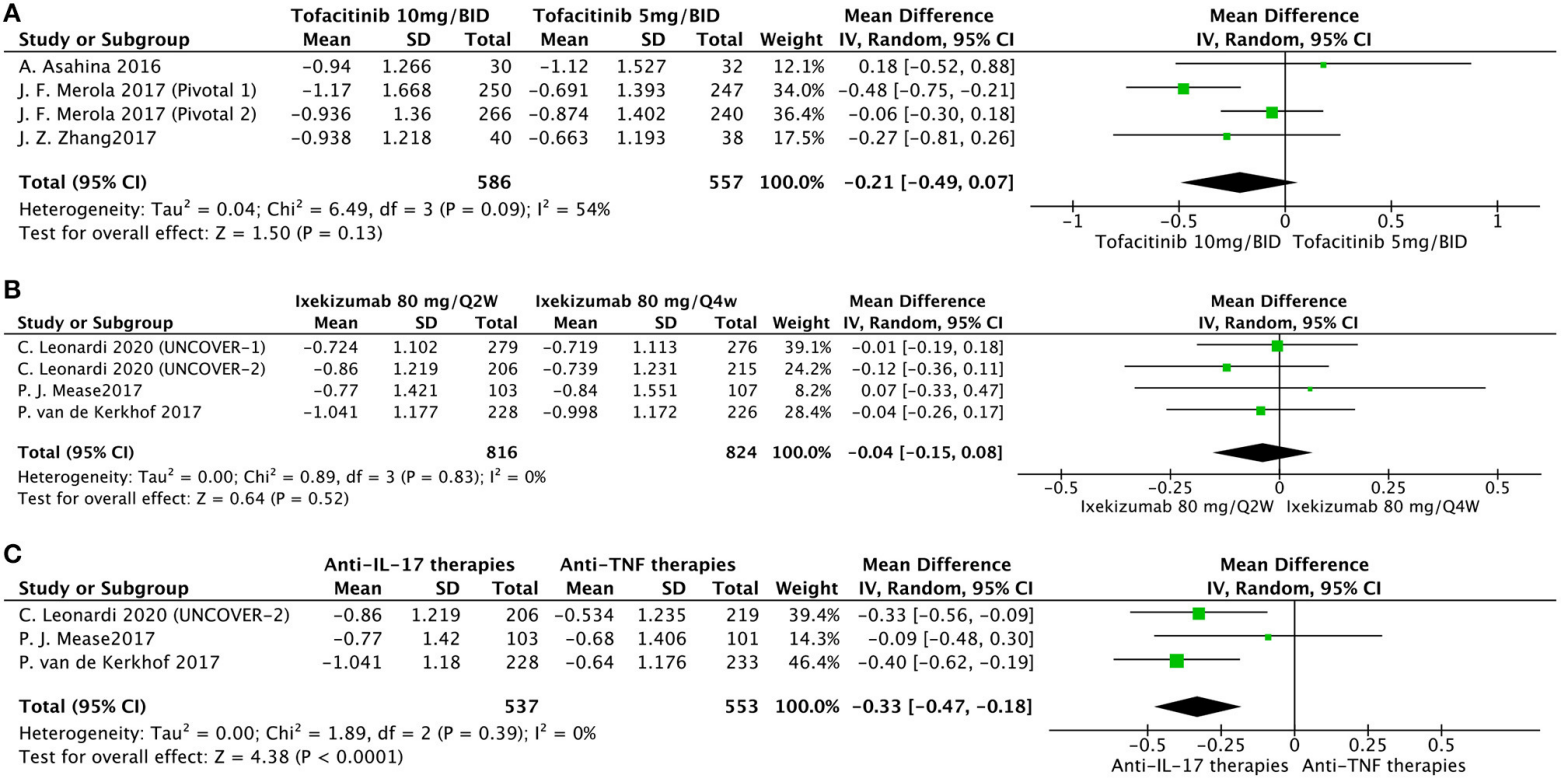
Meta-analysis comparing the effect of multiple intervention groups for the treatment of nail psoriasis. **(A)** tofacitinib 10 mg/BID vs. tofacitinib 5 mg/BID at week 16; **(B)** ixekizumab 80 mg/Q2W vs. ixekizumab 80 mg/Q4W at week 12; **(C)** anti-IL-17 therapies vs. anti-TNF therapies at week 12.

We also conducted other comparisons (Figure 3). Based on available data, we conducted effectiveness comparisons between interventions. Interestingly, a higher dose of tofacitinib did not have a better effectiveness in nail psoriasis at week 16 (Figure 3A). Moreover, Ixekizumab 80 mg/Q2W had a similar outcome in nail psoriasis compared with ixekizumab 80 mg/Q4W at week 12 (Figure 3B). We also found that at week 12, anti-IL-17 therapies were superior to anti-TNF therapies in treating nail psoriasis (Figure 3C).

#### Risk of Bias and Publication Bias Assessment

The included studies were all screened to have a low and unclear risk of bias (Supplementary Figure 3), except in one study (35) where six patients (four in the intervention group and two in the placebo group) dropped out, and the missing data were not imputed. No significant publication bias was detected by using a funnel plot (Supplementary Figure 4) and Egger test (bias, −1.73; 95% CI, −5.16 to 1.70; *P* = 0.298).

## Discussion

This systemic review provides an up-to-date synthesis of published evidence regarding the efficacy of systemic treatments on nail psoriasis and represents a meta-analysis on the efficacy of small-molecule therapies and biologic agents in treating psoriatic nails. In this review, 62.1% of patients with psoriasis had nail involvement, which is consistent with a previous study (1). Nail psoriasis is considered an indicator of systemic immune response (5). One included trial (43) showed that nail psoriasis is associated with a greater PASI, longer course of plaque psoriasis, and a higher proportion of PsA (data not provided). Interestingly, two trials (16, 35) pointed out that the effectiveness of interventions on nail psoriasis is regardless of the presence or absence of PsA. Although PASI scores were not firmly associated with NAPSI scores at baseline, several trials (35, 43, 44, 48) showed that there is a connection between NAPSI and PASI effects during the treatment phase. In general, nail responses were considerably lagged behind cutaneous responses. It's interesting to find out that greater cutaneous responses indicated better nail responses, as the Spearman's correlation between improvements in NAPSI and PASI scores showed a moderate but significant increased over time (35, 48).

Ninety-two percent of the studies included in the systematic review were published after 2010, and majority of trials evaluated small-molecule therapies and biologic agents in psoriasis treatment. They highlighted that available and effective remedies for nail psoriasis have been multiplied in the past decade. However, we noticed that three studies had contradictory outcomes of apremilast in nail psoriasis. Furthermore, one other study (36) unexpectedly reported that ustekinumab failed to provide a significant improvement in NAPSI compared with placebo. Ustekinumab is usually injected subcutaneously at week 0, 4, and then every 12 weeks. It seems unfair for the evaluation of ustekinumab on nail psoriasis that patients received only two doses at week 12 of evaluation.

Relatively few studies were retained in this systematic review evaluating conventional therapies for nail psoriasis and this review also showed their unsatisfied efficacy. This phenomenon was unexpected because acitretin, methotrexate, and cyclosporine play a historical role in systemic psoriasis treatments. However, the available evidence of their efficacy in clinical trials is inadequate, as most studies were either case reports, retrospective or unblinded in design. Anyway, it should be noted that conventional therapies may take a significantly longer time to show improvements in nail psoriasis, which will not be observed by short-term RCTs.

Our meta-analysis emphasized that all evaluated interventions have an eminent beneficial effect in the treatment of nail psoriasis. Tofacitinib showed the most significant scale of effect size in alleviating nail psoriasis (−1.08 points) at week 16. We noticed that the onset of alleviation in nail psoriasis was as early as week 8 in the tofacitinib group (16, 24). The improvement continued throughout the 16 weeks treatment phase. The efficacy of tofacitinib in patients with chronic plaque psoriasis has been previously demonstrated (49). However, one study (50) reported that the tofacitinib 5 and 10 mg/BID groups failed to achieve a significant change in NAPSI compared to the placebo group at month 3 (data not provided). The other therapies also showed significant results: anti-IL-17 (ixekizumab, −0.93 points), anti-TNF (etanercept, adalimumab, infliximab, and golimumab, −0.62 points), and anti-IL-23 (ustekinumab and guselkumab, −0.88 points). The different end timepoints may account for the high heterogeneity between the studies; three studies on week 12 and two studies on week 14 for anti-TNF subgroup analysis (*I*^2^ = 59%) and one on week 12 and one on week 16 for anti-IL-23 subgroup-analysis (*I*^2^ = 75%). We also found that for nail psoriasis, a higher dose of therapies was not the herald of better effectiveness, which is consistent with dose-independent improvement in cutaneous psoriasis, as these therapies may have exceeded the most effective dose (51). Moreover, our meta-analysis showed that anti-IL-17 agents seem to be superior to anti-TNF-α therapies in the treatment of nail psoriasis, consistent with their corresponding effectiveness in cutaneous psoriasis (52).

For patients with psoriatic nails, it was recommended to start with topical anti-psoriatic treatment for at least 4–6 months (13). Conventional systemic therapies were indicated for second-line treatment options for more severe nail psoriasis (13). However, it was also reviewed that these included therapies for cutaneous psoriasis could alleviate coexisting nail disease without noteworthy adverse effects (8). Therefore, the priority of these therapies should be increased for patients with nail psoriasis.

The most important limitation of this meta-analysis is that we could not include all the clinical trials selected in the systematic review because not all of them provided computable changes in the NAPSI score from baseline to the end of the study. Moreover, as variable endpoints (from week 12 to 16), phases (phase II, III) in different studies, and statistical errors due to a relatively small number of patients enrolled in some trials, these results must be displayed meticulously. Also, regarding the slow rate of nail growth to replace the deformed part of the nail plate, the efficacy endpoint for nail evaluation should be optimized in future trials.

Another limitation is that in our systematic review, nearly all of the studies evaluated the effectiveness of interventions on fingernails. One trial (17) showed that the decrease in the toenail NAPSI score is much slower than the fingernail NAPSI score. It is not out of the blue that the average growth rate of the toenails is slower than that of the fingernails, estimated at 1.62 vs. 3.47 mm/month (53). As a result, toenail psoriasis should take a much longer treatment course to achieve the desired outcome.

## Conclusion

In this study, we highlighted that the available biologic therapies and small molecule agents for psoriasis are efficient for nail psoriasis. As nail damage affects more than half of patients with psoriasis, systemic treatment of psoriatic nails should be systematically evaluated in future RCTs as the primary or secondary outcome.

## Data Availability Statement

The raw data supporting the conclusions of this article will be made available by the authors, without undue reservation.

## Author Contributions

XZ and YH: conceptualization. BX: writing. YH: supervision. XZ: software and methodology, data curation. XZ and BX: investigation and formal analysis. All authors contributed to the article and approved the submitted version.

## Conflict of Interest

The authors declare that the research was conducted in the absence of any commercial or financial relationships that could be construed as a potential conflict of interest.
