# Supplementary material for: Efficacy of Systemic Treatments of Nail Psoriasis: A Systemic Literature Review and Meta-Analysis
Source: Front Med (Lausanne). 2021 Feb 10;8:620562. doi: 10.3389/fmed.2021.620562 (PMC7902784; doi:10.3389/fmed.2021.620562)
Supplement: Supplementary file 1 [file Table_1.DOCX]

Supplementary Material

1Supplementary Figures and Tables

## Supplementary Figures


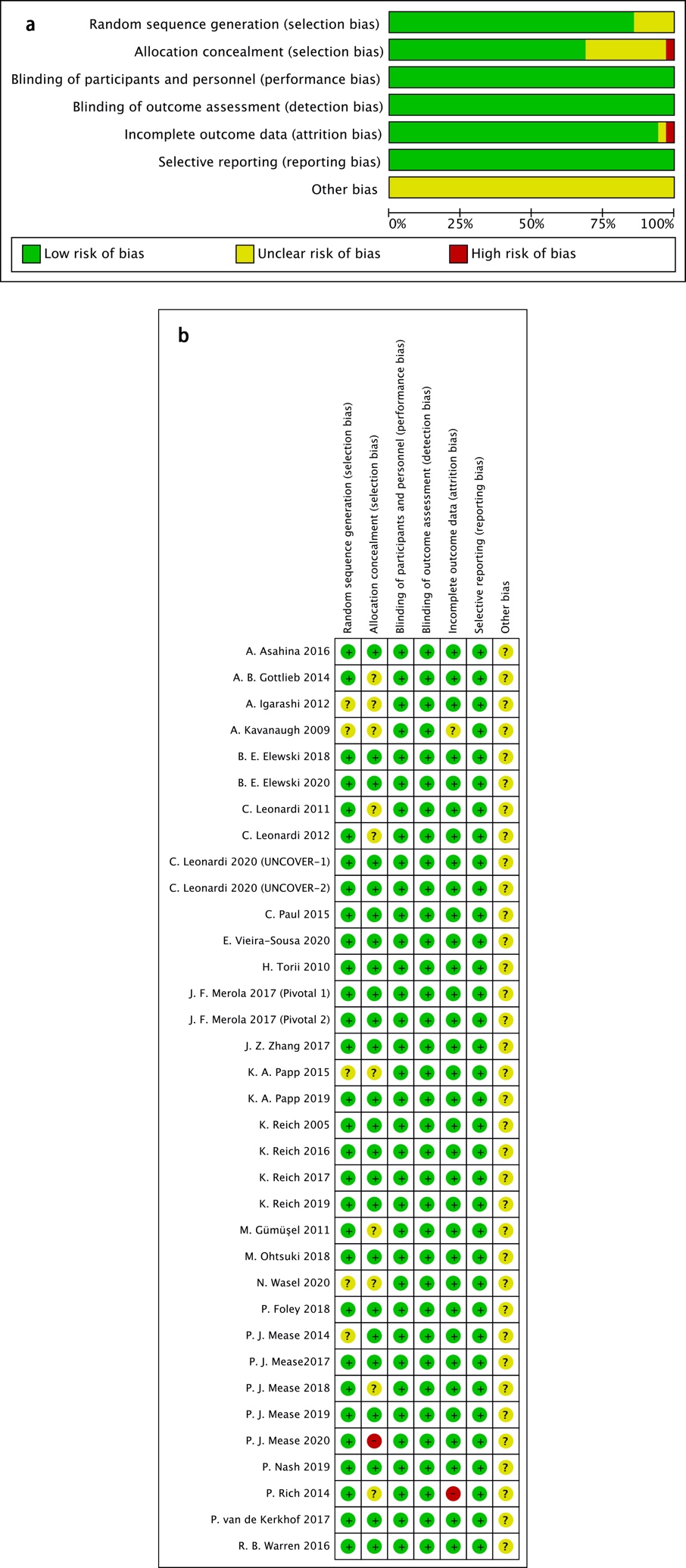


**Supplementary Figure 1.** Risk of bias of included studies for systemic review. a. risk of bias graph; b. risk of bias summary


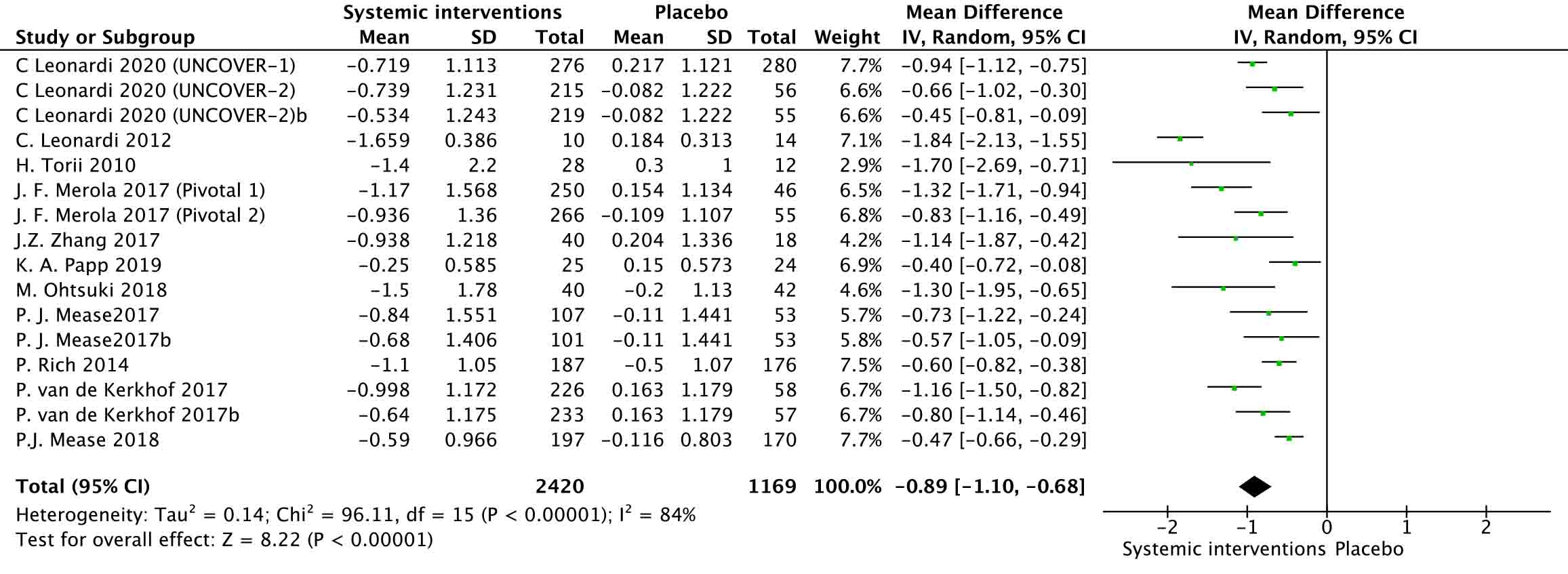


**Supplementary Figure 2.** Global meta-analysis comparing the effect of all systemic treatments vs. placebo for the treatment of nail psoriasis.

**
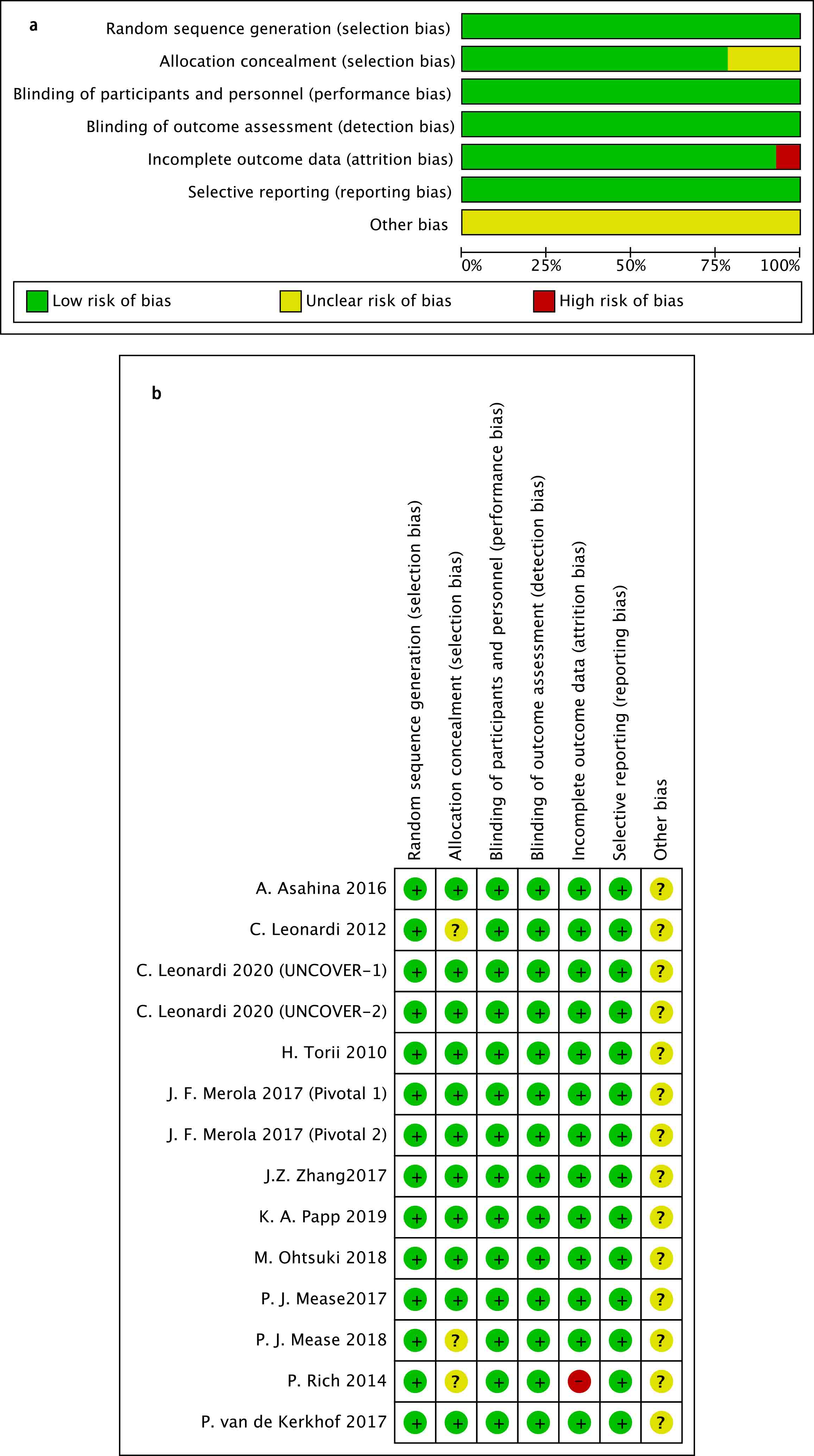
**

**Supplementary Figure 3.** Risk of bias of included studies for meta-analysis. a. risk of bias graph; b. risk of bias summary


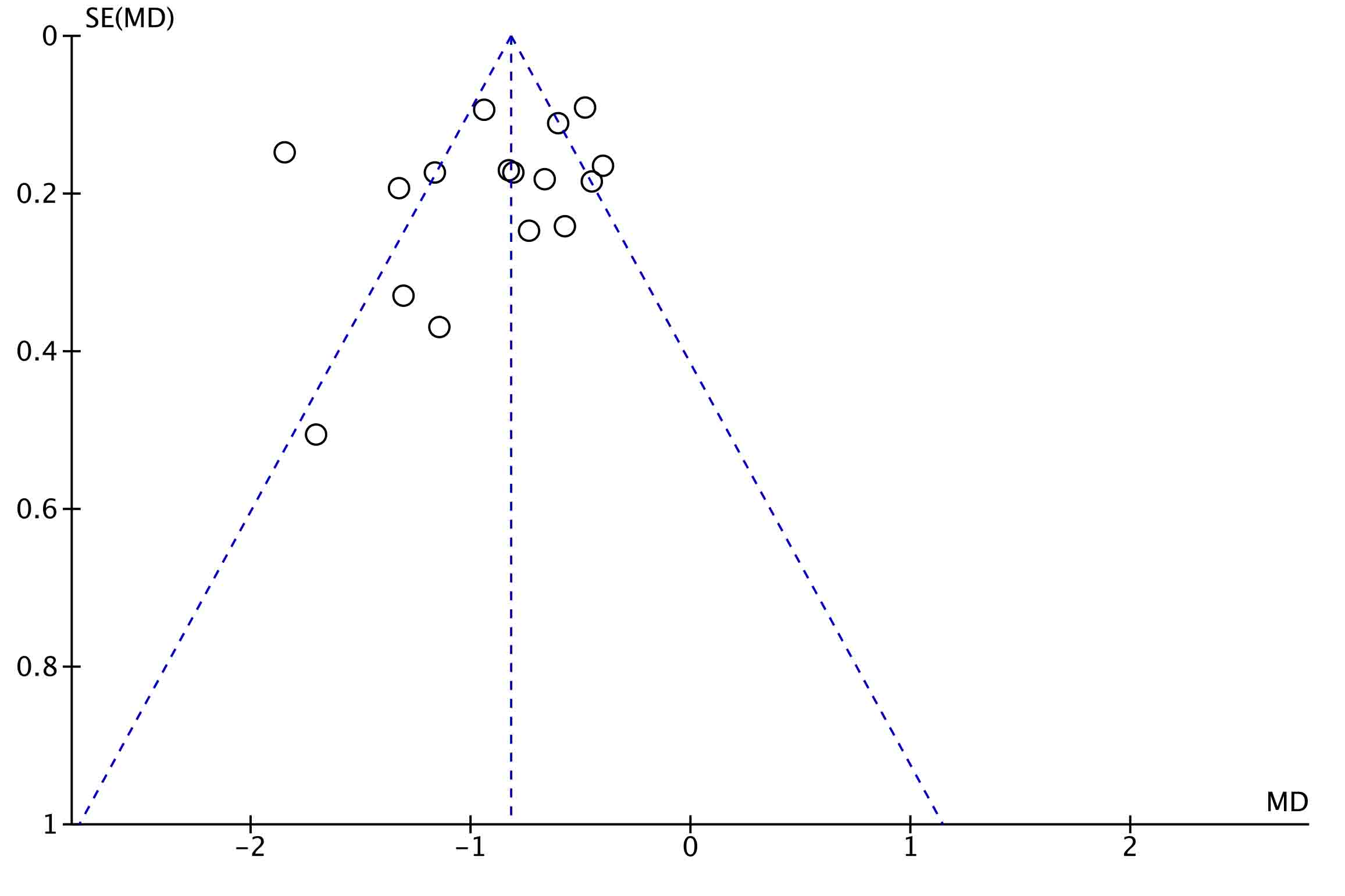


**Supplementary Figure 4.** Funnel plot to detect publication bias.

## Supplementary Tables

STable 1: Summary of Systemic Treatments for Nail Psoriasis from selected studies

| Reference | Treatment | Patients (N)^a^ | Outcome measurement | Baseline NAPSI^b^ | Change in NAPSI score^c^ | P value^d^ |
| --- | --- | --- | --- | --- | --- | --- |
| Small molecule agents – PDE4 inhibitor | | | | | | |
| K. Papp 2015(22) | APR 30 mg/BID | APR (363) PBO (195) | Target fingernail NAPSI (0-8) | 4.3 (2.0) 4.3 (2.16) | Week16 APR -22.5 (54.86)% PBO 6.5 (60.57)% | P < 0.0001 |
| C. Paul 2015(21) | APR 30 mg/BID | APR (175) PBO (91) | Target fingernail NAPSI (0-8) | 4.2 (2.13) 4.4 (2.05) | Week16 APR -29.0%  PBO -7.1% | P=0.052 |
| K. Reich 2017(23) | APR 30 mg/BID ETN 50 mg/QW | APR (50) ETN (50) PBO (42) | Total fingernail NAPSI (0-80) | N/A | Week 16 APR -18.7 (40.2)% ETN-37.3 (45.9)% PBO -10.1 (32.6)% | P=0.495 P=0.002 |
| Small molecule agents – JAK inhibitor | | | | | | |
| J. F. Merola 2017(16) | TOF 5mg/BID or 10mg/BID | TOF 5mg (224) TOF 10mg (229) PBO (102) | Total fingernail NAPSI (0-80) | 26.64 (20.65) 28.24 (20.89) 25.12 (17.27) | Week 16 TOF 5mg -14.15% TOF 10mg -41.48% PBO 55.55% | P < 0.0001 P < 0.0001 |
| J. F. Merola 2017(16) | TOF 5mg/BID or 10mg/BID | TOF 5mg (184) TOF 10mg (175) PBO (104) | Total fingernail NAPSI (0-80) | 27.31 (20.45) 26.22 (20.35) 26.17 (18.63) | Week 16 TOF 5mg -21.62% TOF 10mg -25.97% PBO 15.82% | P < 0.0001 P < 0.0001 |
| J.Z. Zhang 2017(24) | TOF 5mg/BID or 10mg/BID | TOF 5 mg (38)  TOF 10 mg (40) PBO (38) | Total fingernail NAPSI (0-80) | 26.2 (16.97) 25.8 (20.58) 25.2 (16.37) | Week 16 TOF 5 mg BID -14.98%  TOF 10 mg BID -33.32% PBO 7.91% | P=0.1558 P=0.0113 |
| A. Asahina 2016(25) | TOF 5mg/BID or 10mg/BID | TOF 5mg (32) TOF 10mg (34) | Total fingernail NAPSI (0-80) | 29.3 (20.8) 24.3 (17.8) | Week 16 TOF 5mg -11.3 (14.9) TOF 10mg -10.2 (13.2) Week 52 TOF 5mg -20.6 (15.2) TOF 10mg -16.3 (15.3) | N/A |
| Biological agents – Anti-GM-CSF agents | | | | | | |
| K. A. Papp 2019(26) | NAM 20 mg, 50 mg, 80 mg, 150 mg at week 2, 6 and 10 with a loading (double) dose at week 0 | NAM 20 mg (24) NAM 50 mg (24) NAM 80 mg (25) NAM 150 mg (25) PBO (24) | Total fingernail NAPSI (0-80) | 9.6(16.51) 15.6(17.05) 12.0(16.95) 12.5(16.40) 14.5(17.44) | Week 12 NAM 20mg 0.5 (5.78) NAM 50mg -0.9 (5.68) NAM 80mg 15.85) NAM 150mg -1.0 (5.90) PBO 1.5 (5.73) | P=0.537 P=0.142 P=0.015 P=0.121 |
| Biological agents – Anti-TNF-a agents | | | | | | |
| P. J. Mease2019(27) | MTX 20 mg/QW ETN 50 mg/QW | MTX plus ETN (197) MTX plus PBO (185) | Target fingernail mNAPSI (0-8) | 3.6(2.81) 3.4(2.72) | Week 24  MTX plus ETN -1.7 (2.81) MTX plus PBO -1.1 (2.72) | P=0.02 |
| B. E. Elewski 2018(18) | ADA 40mg/Q2W with an initial dose of 80 mg | ADA (109) PBO (108) | Total fingernail NAPSI (range 0-80) | 47.9(16.14) 46.8(15.53) | Week 8 ADA -18.8%  PBO -3.5% Week 26 ADA -54.6%  PBO -14.4% | P<0.001  P<0.001 |
| C. Leonardi 2011(28) | ADA 40mg/Q2W with an initial dose of 80 mg | ADA (28) PBO (8) | Target fingernail NAPSI (0-8) | 3.9(2.0) 3.3(1.8) | Week16 ADA -50% PBO -8% | P=0.02 |
| K. Reich 2005(29) | INF 5 mg/kg at weeks 0, 2, and 6 and every 8 weeks | INF (240) PBO (65) | Target fingernail NAPSI (0-8) | 4.6(2.0) 4.3(1.9) | Week 10 INF -26 (42.3)% PBO 5.9 (54.3)% Week24 INF -56.3 (43.4)% PBO 3.2 (62.3)% | P<0.0001   P<0.0001 |
| H. Torii 2010(30) | INF 5 mg/kg at weeks 0, 2, and 6 and every 8 weeks | INF (29) PBO (14) | Target fingernail NAPSI score (0-8) | 3.7(2.0) 3.5(1.7) | Week 14 INF -1.6 (2.0) PBO 0.6 (0.8) | N/A |
| P. J. Mease 2014(31) | 400 mg CZP at week 0, 2 and 4 followed by either 200mg/Q2W or 400 mg/Q4W | CZP 200mg/Q2W (91) CZP 400mg/Q4W (105) PBO (103) | Target fingernail mNAPSI (0-8) | 3.1(1.8) 3.4(2.2) 3.4(2.2) | Week 24 CZP 200mg/Q2W -1.6 CZP 400mg/Q4W -2.0 PBO -1.1 | P=0.003 P<0.001 |
| A. Kavanaugh 2009(32) | GOL 50mg or 100 mg every 4 weeks | GOL 50mg (95） GOL 100mg (109) PBO (83) | Target fingernail NAPSI (0-8) | 4.7(2.2) 4.6(2.1) 4.8(2.2) | Week 14 (medium) GOL 50mg -25% GOL 100mg -43% PBO 0 | P=0.015 P<0.001 |
| E. Vieira-Sousa 2020(20) | GOL 50mg every 4 weeks | Total 44 patients GOL+MTX (N/A) PBO+MTX (N/A) | Target fingernail NAPSI (0-8) | (medium) 4 4 | Week 12 (medium) GOL+MTX -2  PBO+MTX 0 | P=0.044 |
| P.J. Mease 2018(33) | GOL 2 mg/kg at weeks 0 and 4 and every 8 weeks | GOL (197) PBO (170) | Total fingernail mNAPSI (0-130) | (whole) 18.6 | Week14 GOL -9.6 (15.71) PBO -1.9 (13.05) | P<0.001 |
| B. Elewski 2020(34) | BRD 210 mg every 2 weeks UST 45mg or 90mg stratified by body weight (< 90 kg or ≥ 90 kg) at weeks 0, 4 and every 12 weeks | BRD (104) UST (179) | Target fingernail NAPSI (0-32) | 9.6(4.0) 9.9(3.6) | Week 12 BRD -43.7% UST -31.8%  Week 52 BRD -83.1% UST -75.0% | P<0.05‡  NS‡ |
| Biological agents – Anti-IL-23 agents | | | | | | |
| P. Rich 2014(35) | UST 45 mg or 90 mg at weeks 0, 4, 16 and 28 | UST 45mg (182) UST 90mg (187) PBO (176) | Target fingernail NAPSI (0-8) | 4.5(2.0) 4.4(1.8) 4.3(1.8) | Week12 UST 45mg -26.7 (8.3) % UST 90mg -24.9 (7.1) % PBO -11.8 (7.5)% | P<0.001 P=0.001 |
| A. Igarashi 2012(36) | UST 45 mg or 90 mg at weeks 0, 4, 16 and 28 | UST 45mg (44) UST 90mg (40) PBO (18) | Target fingernail NAPSI (0-8) | 3.7(1.8) 4.1(2.0) 4.6(2.5) | Week12 UST 45mg -7.7 (95.1) % UST 90mg -10.0 (66.1) % PBO 2.9 (27.8)% | P=0.65 P=0.43 |
| M. Ohtsuki 2018(37) | GUS 50 mg, 100 mg at weeks 0, 4 and every 8 weeks | GUS 50mg (44) GUS 100mg (40) PBO (42) | Target fingernail NAPSI (0-8) | 3.8(1.96) 3.7(2.22) 3.6(2.25) | Week 16 GUS 50mg -31.6 (43.56)%  GUS 100mg -39.1 (48.93)% PBO -1.0 (59.38)% | P=0.002 P<0.001 |
| P. Foley 2018(38) | GUS 100mg at week 0, 4 and every 8 weeks ADA 80mg at week 0, 40mg at week 1 and 40mg every 2weeks | GUS (420) ADA (297) PBO (211) | Target fingernail NAPSI (0-8) | 4.8(1.99) 4.5(1.97) 4.9(2.0) | Week 16 GUS -37.5 (44.40)% ADA 41.7 (51.62)% PBO -0.7 (55.44)% | P<0.001 P<0.001 |
| Biological agents – Anti-IL-17 agents | | | | | | |
| K. Reich 2019(17) | SEC 150mg or 300mg, stratified by body weight (< 90 kg or ≥ 90 kg) at baseline and weeks 1, 2, 3 and 4) and every 4 weeks | SEC 300mg (66) SEC 150mg (67) PBO (65) | Total fingernail NAPSI (0-80) | 45.5 (15.6) 39.1 (15.3) 43.2 (15.7) | Week16 SEC 300mg -45.3% SEC 150mg -37.9% PBO-10.8% | p < 0.0001 p < 0.0001 |
| P. Nash 2019(39) | SEC 300 mg at baseline and weeks 1, 2, 3 and 4) and every 4 weeks  loading dose (LD): weeks 1, 2, 3 | SEC 300mg (144) SEC 150mg (135) SEC 150mg (no LD) (153) PBO (231) | Total fingernail NAPSI (N/A) | whole groups 16.4 | Week 16 SEC 300mg -8.71 SEC 150mg -8.95 SEC 150mg (no LD) -7.55 PBO -2.34 | p < 0.0001 p < 0.0001 p < 0.0001 |
| A. B. Gottlieb 2014(40) | SEC 10mg/kg at baseline and weeks 2 and 4, followed by 150 mg or 75 mg every 4 weeks | total 435 patients SEC 75mg (N/A) SEC 150mg (N/A) PBO (N/A) | Total fingernail NAPSI (N/A) | 18.6 18.7 17.5 | Week 24 SEC 75mg -12.3 SEC 150mg -10.9 PBO -4.1 | P < 0.0001 P < 0.0001 |
| C. Leonardi 2012(41) | 10, 25, 75, or 150 mg of IXE at 0, 2, 4, 8, 12, and 16 weeks | IXE 10mg (13) IXE 25mg (10) IXE 75mg (10) IXE 150mg (10) PBO (15) | Total nail NAPSI (0-160) | 46.9 (46.5) 45.0 (46.9) 34.9 (37.7) 41.9 (44.8) 35.0 (28.2) | Week12 IXE 10mg 14.3 (97.8)% IXE 25mg -24.0 (32.8)% IXE 75mg -57.1 (36.7)% IXE 150mg -49.3 (35.9)% PBO 6.8 (41.1)% | NS NS P≤0.01 P<0.05 |
| C Leonardi 2020(15) | 160 mg IXE at baseline followed by 80 mg Q4W or Q2W | IXE Q2w (283) IXE Q4w (281) PBO (283) | Total fingernail NAPSI (0-80) | 24.64 (18.92) 24.12 (18.24) 26.9 (20.49) | Week 12 IXE Q2w -7.24 (11.10)  IXE Q4w -7.19 (11.23) PBO 2.17 (11.27) | P < 0.001 P < 0.001 |
| P. van de Kerkhof 2017(44) | 160 mg IXE at baseline followed by 80 mg Q4W or Q2W ETN 50 mg twice weekly | IXE Q2w (229) IXE Q4w (228) ETN (236) PBO (116) | Total fingernail NAPSI (0-80) | 26.1 (20.1) 26.2 (20.2) 25.1 (20.0) 25.5 (19.6) | Week 12 IXE Q2w -35.2 (90.80)% IXE Q4w -36.7 (90.60)% ETN -20.0 (90.64)% PBO 34.31 (90.47)% | p < 0.001 p < 0.001 p < 0.001 |
| C Leonardi 2020(15) | 160 mg IXE at baseline followed by 80 mg Q4W or Q2W ETN 50 mg twice weekly | IXE Q2W (206) IXE Q4W (215) ETN (219) PBO (111) | Total fingernail NAPSI (0-80) | 26.27 (20.39) 23.7 (18.97) 30.44 (20.65) 27.62 (20.94) | Week 12 IXE Q2W -8.6 (12.20) IXE Q4W -7.39 (12.32) ETN -5.34 (12.43) PBO -0.82 (12.22) | p < 0.001 p < 0.001 p =0.002 |
| P. J. Mease2017(42) | IXE 160 mg at baseline followed by 80 mg Q4W or Q2W INF 40 mg/Q2W | ADA (71) IXE Q4W (70) IXE Q2W (74) PBO (74) | Total fingernail mNAPSI (0-80) | 20.9 (17.5) 21.3 (18.9) 25.0 (21.2) 19.8 (17.2) | Week 12 ADA -6.8 (11.80) IXE Q4W -8.4 (12.55) IXE Q2W -7.7 (12.04) PBO -1.1 (12.04) | P<0.01 P<0.001  P<0.001 |
| N. Wasel 2020(43) | IXE 160 mg at baseline followed by 80 mg/Q2W UST 45mg or 90mg stratified by body weight (< 90 kg or ≥ 90 kg) at weeks 0, 4 and every 12 weeks | IXE (84) UST (105) | Total fingernail NAPSI (0-80) | 24.8 (20.0) 28.3 (19.9) | Week8 UST -2.1 (1.02) IXE-6.6 (1.179) Week52 UST -15.6 (1.12) IXE-22.4 (1.22) | P<0.01‡  P<0.001‡ |
| P. J. Mease 2020(45) | IXE 160 mg at baseline followed by 80 mg/Q4W ADA 40mg/Q2W with an initial dose of 40 mg | IXE (191) ADA (177) | Total fingernail NAPSI (0-80) | 19.7 (18.5) 19.1 (16.3) | Week 24 IXE -15.89 (11.33) ADA -12.53 (10.90) | P=0.001‡ |
| Traditional systemic immunomodulating agents | | | | | | |
| K. Reich 2016(46) | Alitretinoin 30 mg | Alitretinoin (22) PBO (9) | Target fingernail NAPSI (0-8) | 4.8 5.0 | Week 24 Alitretinoin -0.4  PBO -0.4 | NS |
| R. B. Warren 2016(47) | MTX 17.5mg/QW (subcutaneous) | MTX (59) PBO (20) | Target fingernail NAPSI (0-8) | 4.0 4.0 | Week 16 MTX -0.86 (2.02) PBO 0.47 (1.46) | NS |
| M Gümüşel 2011(19) | MTX 15mg/QW CTX 5 mg/kg QD | MTX (18) CTX (19) | Total fingernail NAPSI (0-80) | 39.1(19.9) 42.1(26.4) | Week 24 MTX -43.3% CTX -37.2% | NS‡ |

Abbreviations: PSA, psoriatic arthritis; QD, once a day; QW, once a week; Q2W, every two weeks; Q4W, every four weeks; NAPSI, Nail Psoriasis Severity Index; mNAPSI: modified Nail Psoriasis Severity Index; MTX, methotrexate; CTX, cyclosporine; NAM, namilumab; ETN, etanercept; ADA, adalimumab; INF, Infliximab; CZP, certolizumab pegol; GOL, golimumab; BRD, brodalumab; UST, ustekinumab; GUS, guselkumab; SEC, secukinumab; IXE, Ixekizumab; APR, apremilast; TOF, tofacitinib; PBO: placebo; PDE4: phosphodiesterase 4; JAK: Janus kinase; N/A, data unavailable; NS, P values is not significant.

^a^Data are presented as the number of participants

^b^Data are presented as mean (SD) unless otherwise specified

^c^Data are presented as mean (SD) scores or percentage % unless otherwise specified

^d^P value presented as interventions vs placebo unless otherwise specified

‡ comparisons between interventions
